# Whole mitochondrial genome sequencing in individuals with Leber hereditary optic neuropathy negative for the common pathogenic mitochondrial DNA variants

**DOI:** 10.3389/fneur.2025.1584748

**Published:** 2025-09-01

**Authors:** Sundaramurthy Srilekha, Selvakumar Ambika, Nagarajan Hemavathy, Dharani Vidhya, Patrick Yu-Wai-Man

**Affiliations:** ^1^SNONGC Department of Genetics and Molecular Biology, Medical Research Foundation, Chennai, India; ^2^Department of Neuro-Ophthalmology, Medical Research Foundation, Chennai, India; ^3^Vision Research Foundation, Centre for Bioinformatics, Chennai, India; ^4^John van Geest Centre for Brain Repair and MRC Mitochondrial Biology Unit, Department of Clinical Neurosciences, University of Cambridge, Cambridge, United Kingdom; ^5^Cambridge Eye Unit, Addenbrooke’s Hospital, Cambridge University Hospitals NHS Foundation Trust, Cambridge, United Kingdom; ^6^Moorfields Eye Hospital NHS Foundation Trust, London, United Kingdom; ^7^Institute of Ophthalmology, University College London, London, United Kingdom

**Keywords:** next generation sequencing, homoplasmy, rare variants, bioinformatics analysis, haplogroup analysis

## Abstract

**Purpose:**

This study aimed to explore the role of additional mitochondrial DNA (mtDNA) variants in the development of Leber hereditary optic neuropathy (LHON) by screening the entire mitochondrial genome in individuals who had previously tested negative for the three common mtDNA variants: m.3460G > A (*MT-ND1*), m.11778G > A (*MT-ND4*), and m.14484 T > C (*MT-ND6*), by conventional Sanger sequencing.

**Methods:**

Forty-one individuals with a suspected clinical diagnosis of LHON were recruited from the neuro-ophthalmology clinic. Each participant had undergone a comprehensive neuro-ophthalmic examination, including slit lamp examination, indirect ophthalmoscopy, visual field perimetry, optical coherence tomography, and MRI of the brain and orbits. Targeted re-sequencing was conducted using next-generation sequencing (NGS) on the HiSeqX 10 platform (Illumina, San Diego, California) with a 2 × 150 bp paired-end configuration. The sequencing reads were aligned to the human mitochondrial genome sequence (hg19). Variants were filtered with the VariMAT tool (v.2.3.9). Haplogroup analysis was performed using Haplogrep 2 (v2.0). To assess the deleteriousness of nonsynonymous variations, bioinformatics prediction tools such as PolyPhen2, SIFT, CADD, and Mutation Assessor were utilized. In addition, while tools like Consurf, PredictSNP, DynaMut, ENCoM, DUET, SDM, mCSM, were employed to evaluate evolutionary conservation, pathogenicity, structural stability, and functional impact.

**Results:**

Whole mitochondrial genome sequencing of 41 clinically suspected LHON cases identified a total of 1,518 mtDNA variants. Of these, 822 were located in the coding regions, including 555 synonymous and 273 non-synonymous variants. Two heteroplasmic disease-causing variants (m.11778G > A and m.3460G > A) were identified in one individual each (90.0 and 63.6%, respectively). Additionally, rare mtDNA variants listed in Mitomap were found in five individuals (5/41, 12.1%), namely, *MT-ND1* (m.3335 T > C, m.3394 T > C, m.3395A > G), MT-ND4L (m.10680G > A), and MT-ND6 (m.14502 T > C), with variants in *MT-ND1* being the most prevalent (3/41, 7.3%).

**Conclusion:**

Our study of a well-characterized Indian LHON cohort uncovered rare mtDNA variants that should be considered when assessing undiagnosed optic neuropathy cases. Additionally, it underscores the effectiveness of NGS in identifying heteroplasmic mtDNA variants. This indicates that whole mitochondrial genome sequencing via NGS is a more efficient and preferred approach for routine molecular genetic testing.

## Introduction

Leber Hereditary Optic Neuropathy (LHON; OMIM: 535000) is an inherited optic neuropathy that predominantly affects young adult men (1, 2). LHON is characterized by bilateral subacute loss of central vision loss, either simultaneous at onset as seen in ~25% of cases or sequential within a few weeks of each other (3). It affects about 1:30,000–1:50,000 people (4, 5) with a peak age of onset in the second and third decades of life, but with reports ranging from 2 to 87 years old (6, 7).

The m.3460G > A (A52T), m.11778G > A (R340H), m.14484 T > C (M64V) mitochondrial DNA (mtDNA) variants occurring in the *MT-ND1*, *MT-ND4*, and *MT-ND6* genes, respectively, are the three most common primary mutations that are involved in the pathogenesis of LHON (8–10). Cohort-based studies in various populations found varying proportions of these three mtDNA LHON variants, Denmark −98% (11), Mainland China –68% (12), Japan –100% (13), South Korea −73% (14), Finland −67% (15), and the Northern European –86% (16).

The oxidative phosphorylation (OXPHOS) system, which is located in the inner mitochondrial membrane, consists of five protein complexes and each complex is assembled by multimeric subunit proteins of mitochondrial as well as nuclear origin. The pathophysiology of LHON involves dysfunction of the complex I subunit of the mitochondrial respiratory chain, resulting in increased production of reactive oxygen species and decreased production of ATP.

mtDNA contains 37 genes that encode 13 structural proteins involved in the assembly and function of OXPHOS complexes; 22 tRNAs; and 2 rRNAs together involved in mitochondrial protein synthesis (17). LHON is caused by mtDNA variants that disrupt the protein complexes of the OXPHOS systemoften occurring in the genes encoding the subunits of complex I, the NADH: ubiquinone oxidoreductase. In addition to the three common LHON mtDNA variants, 16 other pathogenic variants have been reported^1^ that account for <5% of LHON cases. Previous studies indicate that specific mitochondrial haplogroups and their subgroups influence the pathogenicity and disease penetrance of the three common LHON mtDNA variants (18). A meta-analysis of 3,613 subjects from 159 white Caucasian LHON pedigrees, showed that the risk of visual failure is greater when the m.11778G > A and m.14484 T > C variants are present in specific subgroups of haplogroup J (J2 for m.11778G > A and J1 for m.14484 T > C), and when the m.3460G > A variants is present in haplogroup K (18). In the Chinese Han population, the M7b1’2 haplogroup increased the risk of visual loss whereas the M8a haplogroup had a protective effect (19–21). In the Indian population, the m.11778G > A and m.14484 T > C variants did not demonstrate significant haplogroup associations (22, 23). The M haplogroup was found to be the most common in both LHON cases and healthy controls in another South Indian study (24).

A reduced frequency of the three common LHON mutations has been identified in the North (27.5%) (25) and South Indian (43.6%) (26) populations. We have previously reported a figure of 29.4% in a combined North and South Indian population (27) emphasizing that the Indian population may exhibit a different mutational LHON profile with the involvement of pathogenic variants other than m.3460G > A, m.11778G > A and m.14484 T > C. In this study, we have further explored these pathogenic variants by analyzing the entire mitochondrial genome in a cohort of individuals with a clinical diagnosis of LHON. Phylogenetic analysis was also carried out to investigate the association of specific mtDNA haplogroups with LHON in the North and South Indian populations, and to predict the group of carriers at higher risk of developing visual loss.

## Materials and methods

### Clinical examination

The study protocol was approved by the Institutional Review Board (IRB) and ethics committee, and all procedures were performed according to institutional guidelines and the Declaration of Helsinki. Study participants underwent detailed ophthalmic examination by an experienced neuro-ophthalmologist (SA) and her team, including slit lamp examination, indirect ophthalmoscopy, visual field perimetry, optical coherence tomography, and MRI of the brain and orbits. Visual field analysis was performed according to the Humphrey automated visual field analyzer Swedish Interactive Threshold Algorithm (SITA) 30–2 standard. A diagnosis of LHON was based on classical clinical features, namely, a young patient with bilateral subacute progressive central visual loss, color vision defects, and a central or centrocecal scotoma, following exclusion of other acquired causes. These cases were referred for genetic screening.

### DNA isolation and primary mutation screening

In patients referred for genetic testing, a detailed pedigree was taken, followed by blood collection for DNA extraction. Genomic DNA was extracted using a Nucleospin blood XL kit (Macherey-Nagel, GmbH, Düren, Germany) according to the manufacturer’s instructions. The three common LHON mtDNA mutations were screened according to the protocol used for our previous study (27).

### Next generation sequencing of whole mitochondrial genome

Library preparation and purity were performed according to the manufacturer’s protocol. Briefly PCR products were amplified (Qiagen long-range PCR kit) with MTL-F1 5′-AAA GCA CAT ACC AAG GCC AC-3′ MTL-F2 5′- TAT CCG CCA TCC CAT ACA TT-3′ MTL-R1 5′-TTG GCT CTC CTT GCA AAG TT-3′ MTL-R2 5′-AAT GTT GAG CCG TAG ATG CC-3′ these sets of specific primers and fragments pooled together for NGS library preparation. These two fragments were pooled equally for NGS library preparation. Firstly, the DNA was fragmented (~250 bp), repairing ends, adenylation of 3’ends, followed by adapter ligation. At each step the products were purified using AMPure beads (Beckman Coulter). The adapter sequences were added onto the ends of DNA fragments to generate paired-end libraries. The resulting adaptor-ligated libraries were purified, and index tags were added by amplification, followed by purification. Libraries were assessed to check the quality and quantity by using an (Agilent 2200 Tape station).

Prepared libraries were quantified using the Qubit HS Assay (Invitrogen). The obtained libraries were pooled and diluted to the final optimal loading concentration (2 nm in 10 μL) before cluster amplification on an Illumina flow cell. Once the cluster generation was completed, the cluster flow cell was loaded on the Illumina HiSeq X instrument following the manufacturer’s instructions (Illumina) to generate 2*150 bp paired-end reads at >500X sequencing depth.

### Sequence data analysis

The coverage and depth were analyzed after the quality check and alignment to the human mitochondrial genome (hg19). Alignment for all samples was in the range of 94–99%. The average depth for all samples was above 10,000X. GATK version 3.6 (28) was used for calling germline variants and annotated using the in-house VariMAT program (v.2.3.9). It integrates multiple clinical-grade databases, variant class prediction, and variant pathogenicity prediction tools for annotating the variants that rely on the Variant effect predictor [VEP] (29). Some of the annotated information by VariMAT was from population frequency, computational pathogenicity prediction, variant type, and predicted impact of the variant on the protein (e.g., missense and loss of function). A total sequencing coverage of more than 10,000X of the mitochondrial genome was achieved across all the samples. At such coverage, heteroplasmy at 5% levels would be detectable. We determined whether the variations were Novel, Reported, or with any disease association by investigating their presence in the MITOMAP, Human Mitochondrial Genome Database (HmtDB), ClinVar databases, Genome Aggregation Database (GnomAD v3.1.1), as well as Google and PubMed searches for individual studies reporting mtDNA variations. Haplogroup classification of mitochondrial samples was done by using the Haplogrep3 tool (30).

### Bioinformatics analysis

#### Conservation and functionality analysis of variants

Bioinformatics predictions were conducted for missense variants that lack pathogenicity data in ClinVar, as well as for variants classified as Variants of Unknown Significance (VUS) and helper variants. These variants were first subjected to conservation analysis using ConSurf (31) to assess the evolutionary conservation of the affected amino acids and predict their functional significance. Subsequently, sequence-based pathogenicity, structural stability, and functional impact were evaluated using multiple prediction tools, including PredictSNP (32), MAPP Prediction (33), PhD-SNP Prediction (34), PolyPhen-1 (35), PolyPhen-2 (36), and SIFT (37). To further examine the effect of these variants on protein structural stability, *in silico* tools such as DynaMut (38), ENCoM (39), DUET, SDM (40), and mCSM (41) were employed. In addition, the MitImpact 3D tool (55),^2^ which exclusively provides insights into the genetic and clinical implications of the non-synonymous substitutions in human mitochondrial protein-coding genes, was also used to determine the conservation and pathogenicity of the variants. Prior to stability analysis, Alphafold-derived protein structures were retrieved and refined. Variants were generated using PyMOL, followed by refinement and preprocessing of both wild-type and variant forms with the Schrodinger suite. Finally, the potential impact of these variants on protein functionality was assessed using the MutPred2 server (42).

## Results

### Demographics and clinical data

This study included 41 unrelated probands who were negative for the three primary LHON mutations based on sanger sequencing. The median age at disease onset was 20.2 years (standard deviation 10.6 years), with a range from 1 to 45 years. All patients had either diffuse or temporal optic disk pallor on fundus examination. The clinical characteristics and examination findings have been summarized in Supplementary Table 1.

### Next generation sequencing of whole mitochondrial genome

**Figure 1:**
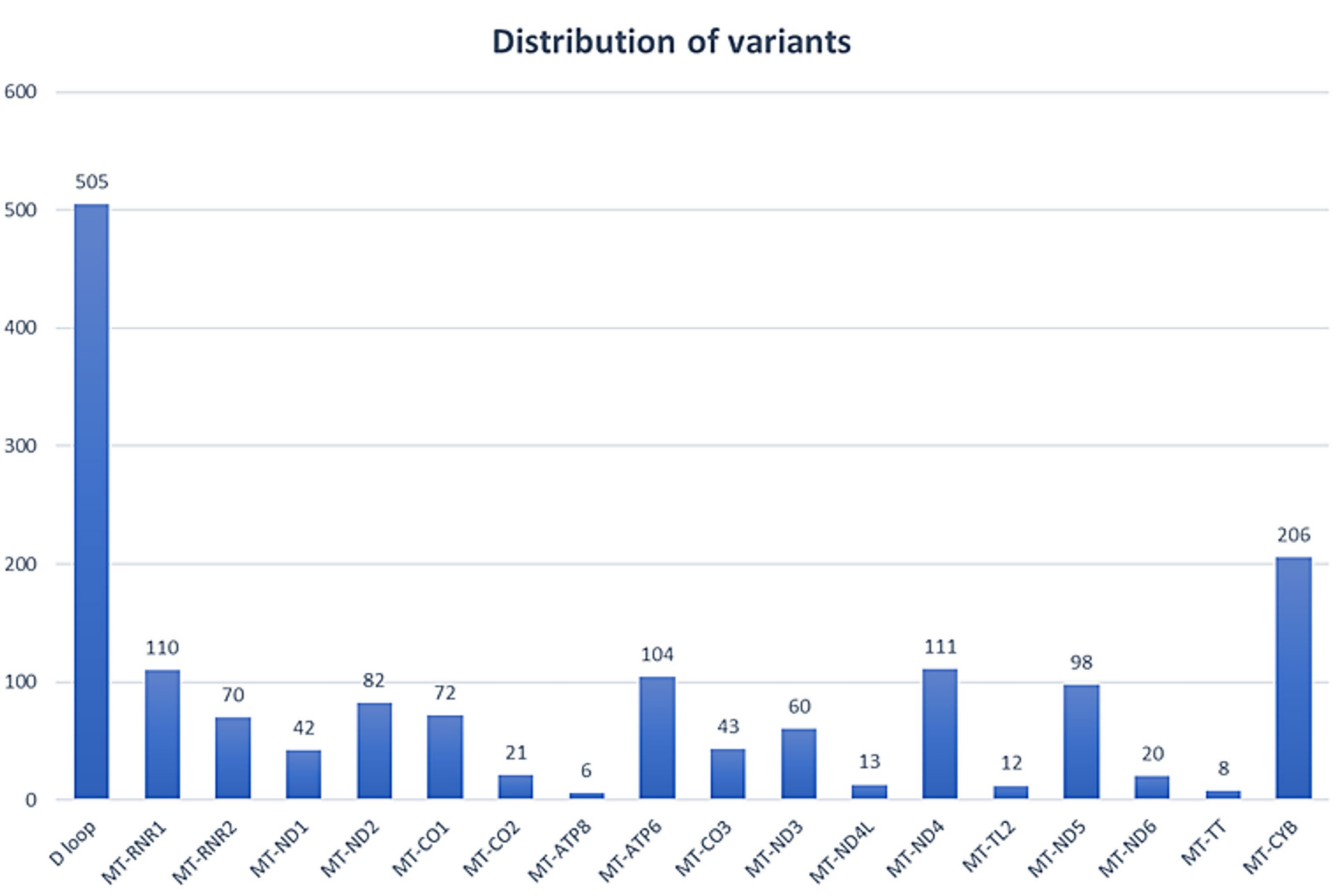
Distribution of variants across the mitochondrial genome.

**Figure 2:**
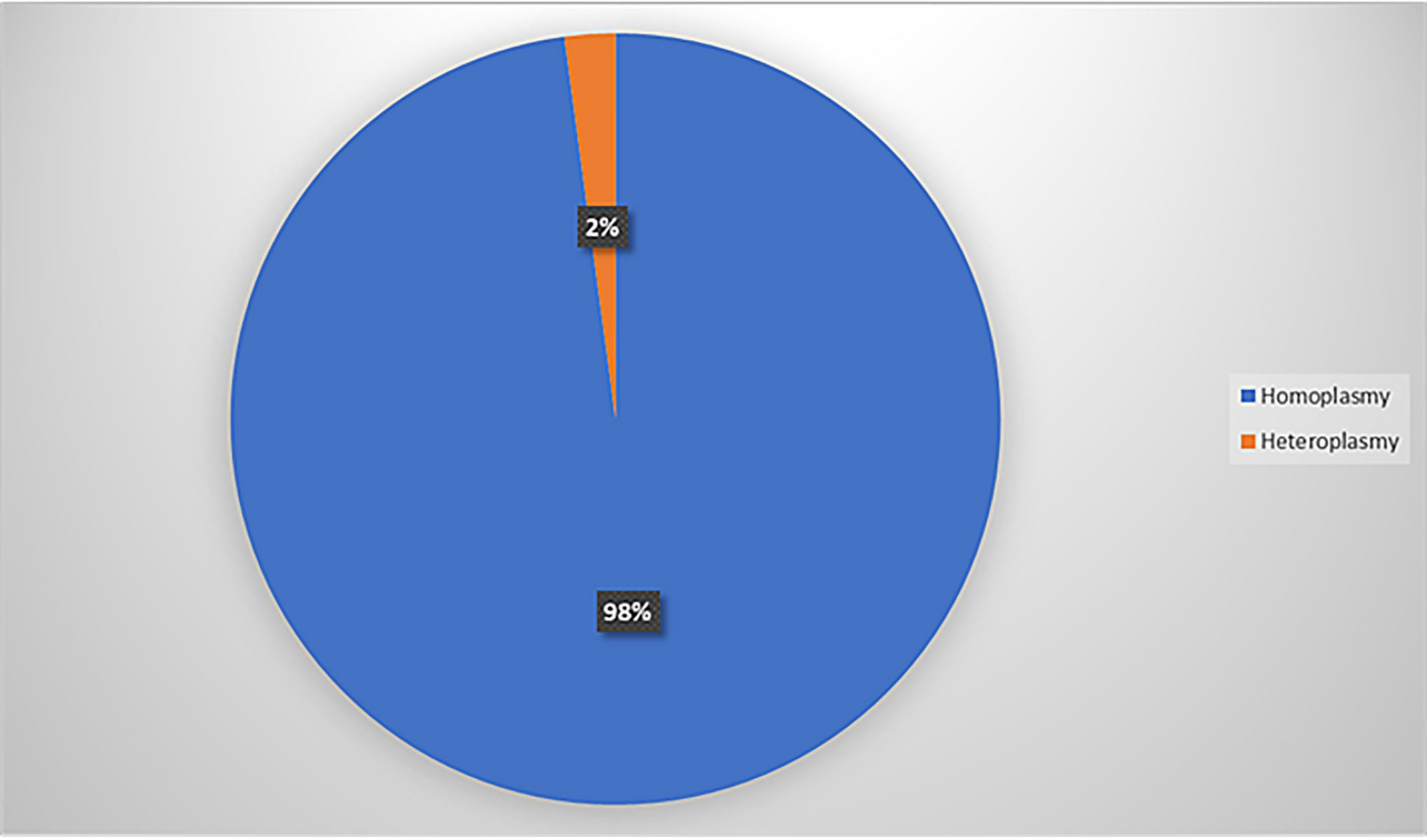
Distribution of homoplasmic and heteroplasmic variants.

A comprehensive analysis of the whole mitochondrial genome was conducted for 41 individuals using NGS, with all samples meeting quality control standards. Our findings revealed a widespread distribution of variants across the mitochondrial genome, particularly in the D-loop region (Figure 1), and with most (98%) being homoplasmic (Figure 2). Detailed case-wise distributions of missense variants observed in suspected LHON cases have been provided in Supplementary Table 2. Among the probands, two individuals who were previously found to be negative for the three primary mutations via Sanger screening were found to be positive for m.11778G > A and m.3460G > A in a heteroplasmic state (90.0 and 63.6%, respectively) through NGS (2/41).

Rare pathogenic variants were reported in five probands: *MT-ND1* (3/41, 7.3%), *MT-ND4L1* (1/41, 2.4%), and *MT-ND6* (1/41, 2.4%) are listed in Table 1. They are *MT-ND1* (m.3335 T > C, m.3394 T > C, m.3395A > G), *MT-ND4L* (m.10680G > A), and *MT-ND6* (m.14502 T > C). Additionally, two probands shared the same helper variant in *MT-CO3* (2/41, 4.8%). The overall mutation detection rate in suspected LHON cases was 17.0% (7/41). Notably, we identified seven cases with variants classified as having conflicting interpretations of pathogenicity in *MT-ND1* and *MT-ND2* (Table 2). Furthermore, common missense variants in *MT-ATP6*, *MT-ND3*, and *MT-CYB* were observed that are present at high frequencies in GenBank, as referenced in MITOMAP and GnomAD (Table 3).

**Table 1:** Rare pathogenic variants identified in the study.

S. no	Sample no	Age/sex	Age of onset	Levels of visual impairment	Neuroimaging (MRI) brain/orbit	Rare pathogenic variants
1.	Sample 9	29/M	24	Moderate	Normal	G10680A
2.	Sample 29	14/M	11	Moderate	Normal	T3355C
3.	Sample 36	27/M	27	Moderate	Mild T2 hyperintensities with mild diffusion restriction involving the bilateral optic nerve sheath complexes (R > L)	T3394C
4.	Sample 39	28/M	15	Severe	Cisternal segment of the left 6th nerve is thinned out	A3395G
5.	Sample 47	22/M	13	Moderate	Normal	T14502C

**Table 2:** Conflicting interpretations of pathogenicity.

Gene	Mutation	REF	ALT	cDNA	Variation	Amino acid	Zygosity	Genbank frequency (GB) (Mitomap) %	GnomAD allele frequency%	Clinvar	Sample nos
*MT-ND1*	m.3316	G	A	c.10G > A	Missense	p. A4T	Homozygous	R30a1b1	0.093	Benign	4
*MT-ND1*	m.4216	T	C	c.910 T > C	Missense	p. Y304H	Homozygous	R2d	10.236	Benign	11,13,16
*MT-ND2*	m.5460	G	A	c.991G > A	Missense	p. A331T	Homozygous	M58	6.754	Benign	36,41,53

**Table 3:** Frequently observed Benign missense variants with high GB and GnomAD frequency.

Gene	Mutation	REF	ALT	cDNA	Variation	Amino acid	Zygosity	Genbank frequency (GB) (Mitomap) %	GnomAD allele frequency%	Clinvar	Sample nos
*MT-ATP6*	m.8701	A	G	c.175A > G	MISSENSE	p. T59A	Homozygous	31.5	30	Benign	3,6,9,12,16.19,21,23,24,26,27,34,35,37,38,39,40,41,42,46,48,50
*MT-ATP6*	m.8860	A	G	c.334A > G	MISSENSE	p. T112A	Homozygous	98.4	99	Benign	3,6,8,9,11,12,13,16,18,19,21,22,23,24,25,26,27,28,29,30,33,34,35,36,37,38,39,40,41,42,43,44,45,46,47,48,50,51,52,53
*MT-ND3*	m.10398	A	G	c.340A > G	MISSENSE	p. T114A	Homozygous	42.5	41	Benign	3,6,9,12,16,19,21,23,24,26,27,34,35,37,38,39,40,41,42,46,48,50,51,52,53
*MT-CYB*	m.14766	C	T	c.20C > T	MISSENSE	p. T7I	Homozygous	75.6	70	Benign	3,6,8,9,11,12,13,16,18,19,21,22,23,24,25,26,27,28,29,30,33,34,35,36,37,38,39,40,41,42,43,44,45,46,47,48,50,51,52,53
*MT-CYB*	m.15326	A	G	c.580A > G	MISSENSE	p. T194A	Homozygous	98.5	99	Benign	3,6,8,9,11,12,13,16,18,19,21,22,23,24,25,26,27,28,29,33,34,35,36,37,38,39,40,41,42,43,44,45,46,47,48,50,51,52,53

### Conservation and functionality analysis of variants

**Figure 3:**
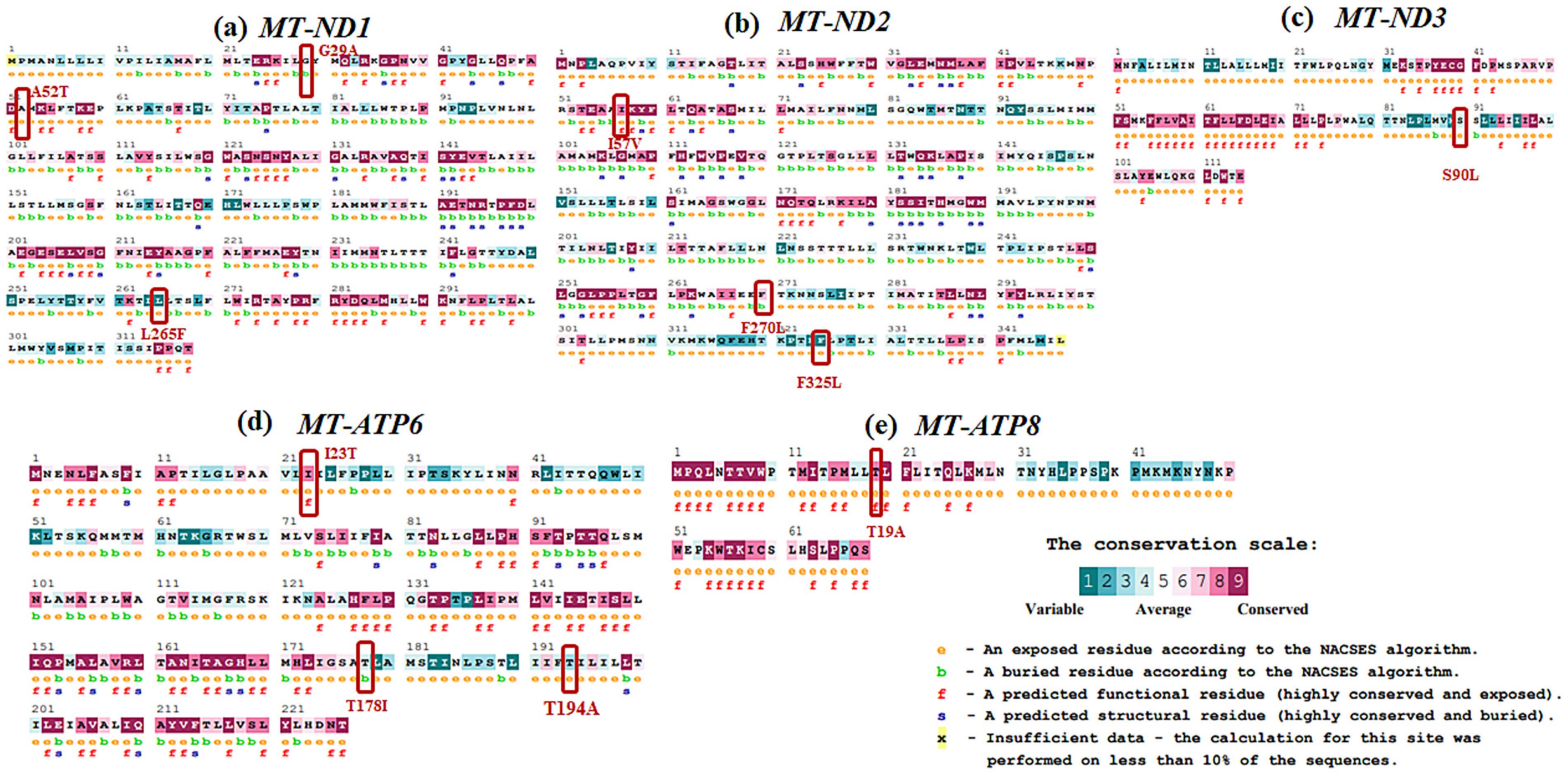
Evolutionary conservation analysis of **(a)** *MT*-*ND1*, **(b)** *MT*-*ND2*, **(c)** *MT*-*ND3*, **(d)** *MT*-*ATP6*, **(e)** *MT*-*ATP8* with the positions of the variants highlighted in boxes.

**Figure 4:**
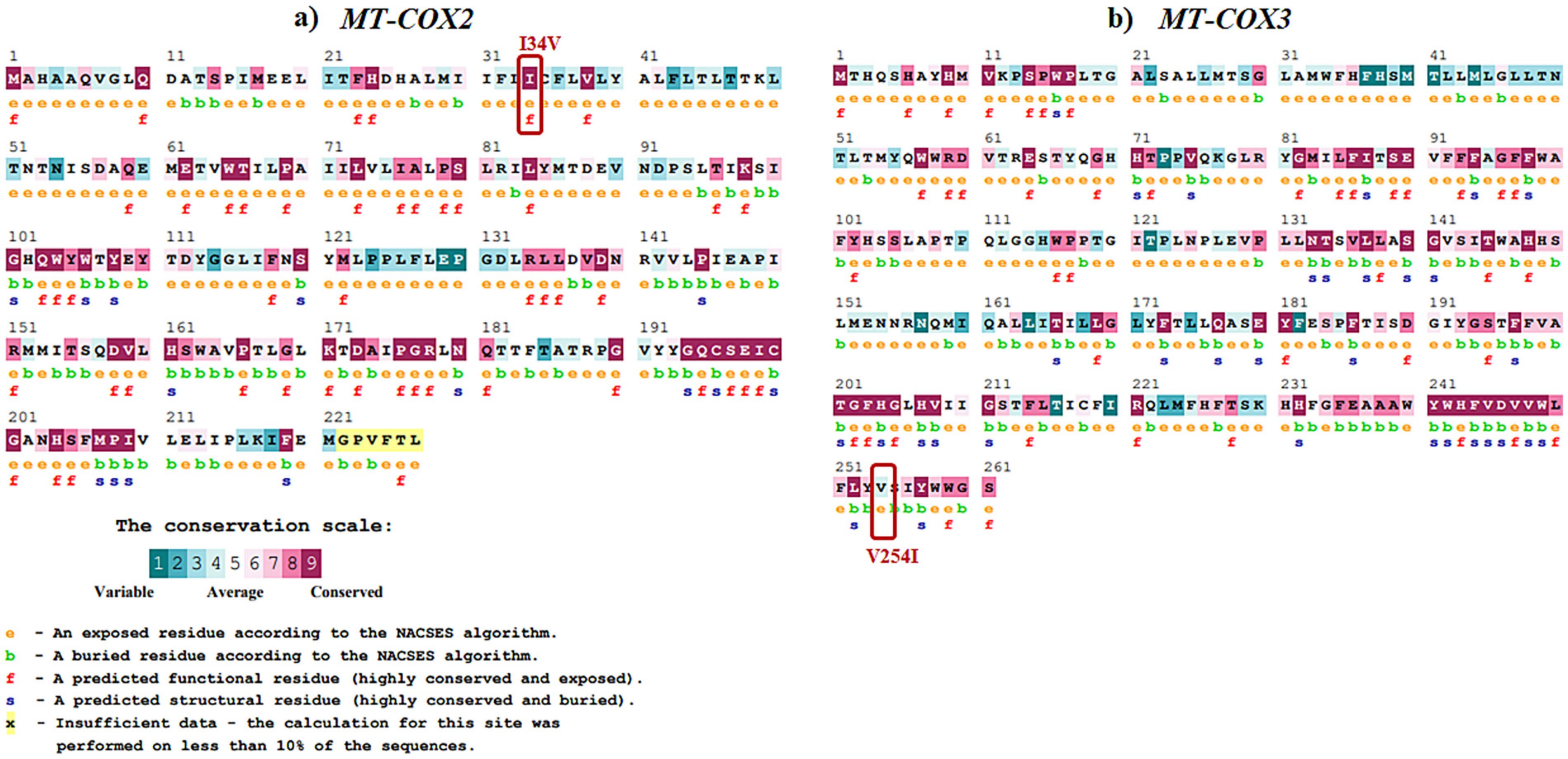
Evolutionary conservation analysis of **(a)** *MT*-*COX2* and **(b)** *MT*-*COX3* with the positions of the variants highlighted in boxes.

Evolutionary conservation analysis was conducted using ConSurf (Table 4; Figures 3, 4). For *MT-ND1* (Figure 3A), residues m.3316G > A (p. A4T). m.4099C > T (p. L265F), show the least conservation and are exposed, with m.3392G > C (p. G29A) first substitution, and m.3460G > A (p. A52T) having average conservation. For *MT-ND2* (Figure 3b), residues m.4638A > G (p. I57V) and m.5279C > A (p. F270L) are highly conserved, but only m.4638A > G (p. F57V) has functional implications. For *MT-ND3* (Figure 3c), m.10327C > T (p. S90L) shows the least conservation. For *MT-ATP6* (Figure 3d), residue m.8594 T > C (p. I23T) is highly conserved and predicted to have functional impact, while residues m.9059C > T (p. T178I), and m.9106A > G (p. T194A) are least conserved and buried, suggesting that variants at these tyrosine residues will not significantly affect the protein’s structure or function. For *MT-ATP8* (Figure 3e), residue m.8420A > G (p. T19A) is highly conserved. For *MT-CO2* and *MT-CO3*, residue m.7685A > G (p. I34V) in *MT-CO2* exhibits greater conservation compared to the least conserved residues in *MT-CO3* (Figures 4a,b).

**Table 4:** Conservation analysis of all the variants using Consurf.

S. no	Sample ID	Gene	Variant	Amino acid	Wild residue	Position	Target residue	Conservation score	S/F
1.	Sample 50	*MT-ND1*	m.3392G > C	G29A	G	29	A	6	B
2.	Sample 50	*MT-ND1*	m.3460G > A	A52T	A	52	T	7	E
3.	Sample 27,49	*MT-ND1*	m.4099C > T	L265F	L	265	F	1	E
4.	Sample 40	*MT-ND2*	m.4638A > G	I57V	I	57	V	8	F/E
5.	Sample 34	*MT-ND2*	m.5444C > A	F325L	F	325	L	8	E
6.	Sample 3	*MT-ND2*	m.5279C > A	F270L	F	270	L	7	B
7.	Sample 50	*MT-ND3*	m.10327C > T	S90L	S	90	L	3	E
8.	Sample 35	*MT-ATP8*	m.8420A > G	T19A	T	19	A	8	F/E
9.	Sample18, 44, 47	*MT-ATP6*	m.8594 T > C	I23T	I	23	T	8	F/E
10.	Sample 12	*MT-ATP6*	m.9059C > T	T178I	T	178	I	4	B
11.	Sample 49	*MT-ATP6*	m.9106A > G	T194A	T	194	A	3	E
12.	Sample 27,49	*MT-CO3*	m.9966G > A	V254I	V	254	I	4	E
13.	Sample 28	*MT-CO2*	m.7685A > G	I34V	I	34	V	9	F/E

From the conservation prediction using MitImpact 3D, of the 13 variants, only five variants occurring at amino acid positions have been observed to be highly conserved. In specific, the *MT-ND1* variants, m.3392G > C and m.3460G > A, and other gene variants namely, *MT-ND2* (m.4638A > G), *MT-ATP6* (m.8594 T > C), *MT-CO2* (m.7685A > G) were observed to be highly conserved in all the predictors except for PhastCons 470way predictor (Supplementary Table 3).

Overall, from the consensus analysis of all the predictors, it was observed that the amino acid positions of the following variants, namely, m.4099C > T (*MT-ND1*), m.5444C > A (*MT-ND2*), m.10327C > T (*MT-ND3*), m.9059C > T and m.9106A > G (*MT-ATP6*), m.9966G > A (*MT-CO3*) are non-conserved.

### Pathogenicity analysis and structural stability analysis

The m.8594 T > C variant in *MT-ATP6* was identified as highly deleterious by all bioinformatics tools (Table 5). In terms of the meta-predictors, it was observed that the *MT-ND1* variants (m.3392G > C and m.3460G > A) were observed to be damaging and deleterious among the five meta-predictors. In case of APOGEE1-based prediction, only *ND1* variants (m.3392G > C and m.3460G > A) and *MT-CO2* variant m.7685A > G are pathogenic (Supplementary Table 4).

**Table 5:** Pathogenicity analysis of the mitochondrial DNA variants.

S. no	Sample ID	Gene	Variant	Amino acid	PredictSNP prediction	PredictSNP expected accuracy	MAPP prediction	MAPP expected accuracy	PhD-SNP prediction	PhD-SNP expected accuracy	PolyPhen-1 prediction	PolyPhen-1 expected accuracy	PolyPhen-2 prediction	PolyPhen-2 expected accuracy	SIFT prediction	SIFT expected accuracy
1.	Sample 18, 44, 47	MT-ATP6	m.8594 T > C	I23T	DELETERIOUS	0.719	DELETERIOUS	0.427	DELETERIOUS	0.773	DELETERIOUS	0.594	DELETERIOUS	0.551	DELETERIOUS	0.793
2.	Sample 12	*MT-ATP6*	m.9059C > T	T178I	NEUTRAL	0.603	NEUTRAL	0.705	NEUTRAL	0.719	DELETERIOUS	0.594	DELETERIOUS	0.503	DELETERIOUS	0.793
3.	Sample 49	*MT-ATP6*	m.9106A > G	T194A	NEUTRAL	0.753	NEUTRAL	0.717	NEUTRAL	0.832	NEUTRAL	0.669	NEUTRAL	0.703	DELETERIOUS	0.793
5.	Sample 35	*MT-ATP8*	m.8420A > G	T19A	NEUTRAL	0.748	DELETERIOUS	0.877	NEUTRAL	0.892	NEUTRAL	0.669	NEUTRAL	0.644	NEUTRAL	0.7
6.	Sample 28	*MT-CO2*	m.7685A > G	I34V	NEUTRAL	0.603	DELETERIOUS	0.718	NEUTRAL	0.892	NEUTRAL	0.669	NEUTRAL	0.644	DELETERIOUS	0.793
7.	Sample 27,49	*MT-CO3*	m.9966G > A	V254I	NEUTRAL	0.826	NEUTRAL	0.851	NEUTRAL	0.832	NEUTRAL	0.669	NEUTRAL	0.763	NEUTRAL	0.671
8.	Sample 50	MT-ND1	m.3392G > C	G29A	DELETERIOUS	0.607	DELETERIOUS	0.771	NEUTRAL	0.508	DELETERIOUS	0.594	DELETERIOUS	0.65	DELETERIOUS	0.793
9.	Sample 27,49	MT-ND1	m.4099C > T	L265F	NEUTRAL	0.653	DELETERIOUS	0.409	NEUTRAL	0.832	NEUTRAL	0.669	NEUTRAL	0.723	DELETERIOUS	0.793
10.	Sample 50	*MT-ND1*	m.3460G > A	A52T	DELETERIOUS	0.756	DELETERIOUS	0.842	DELETERIOUS	0.589	DELETERIOUS	0.745	DELETERIOUS	0.811	DELETERIOUS	0.528
12.	Sample 40	*MT-ND2*	m.4638A > G	I57V	NEUTRAL	0.632	DELETERIOUS	0.484	NEUTRAL	0.719	NEUTRAL	0.669	NEUTRAL	0.725	DELETERIOUS	0.793
13.	Sample 3	*MT-ND2*	m.5279C > A	F270L	NEUTRAL	0.738	NEUTRAL	0.796	NEUTRAL	0.832	NEUTRAL	0.669	NEUTRAL	0.763	DELETERIOUS	0.459
14.	Sample 34	*MT-ND2*	m.5444C > A	F325L	NEUTRAL	0.826	NEUTRAL	0.796	NEUTRAL	0.783	NEUTRAL	0.669	NEUTRAL	0.873	NEUTRAL	0.682
15.	Sample 50	*MT-ND3*	m.10327C > T	S90L	NEUTRAL	0.737	NEUTRAL	0.65	NEUTRAL	0.661	DELETERIOUS	0.594	NEUTRAL	0.789	NEUTRAL	0.775

Structural stability analysis of the refined structures (Supplementary Figures 1–5) indicated that the variants in *MT-ND1, MT-ND2, MT-ATP6,* and *MT-CO3* led to structural destabilization (Table 6). Specifically, the variants m.8594 T > C (*MT-ATP6*) and m.5279C > A (*MT-ND2*), m.7685A > G (*MT-CO2*), were found to negatively impact their respective protein structures.

**Table 6:** Structural stability analysis of the identified mitochondrial DNA variants.

S. no	Sample ID	Gene	Variant	Amino acid	ΔΔG DynaMut (kcal/mol)	ΔΔG ENCoM (kcal/mol)	ΔΔG mcsm (kcal/mol)	ΔΔG SDM (kcal/mol)	ΔΔG DUET (kcal/mol)
1.	Sample 12	*MT-ATP6*	m.9059C > T	T178I	1.178 (Stabilizing)	0.367 (Destabilizing)	−0.546 (Destabilizing)	1.200 (Stabilizing)	0.084 (Stabilizing)
2.	Sample 49	*MT-ATP6*	m.9106A > G	T194A	0.438 (Stabilizing)	−0.015 (Destabilizing)	−0.686 (Destabilizing)	1.980 (Stabilizing)	0.136 (Stabilizing)
3.	Sample 18, 44, 47	*MT-ATP6*	m.8594 T > C	I23T	−1.240 (Destabilizing)	−0.185 (Destabilizing)	−1.854 (Destabilizing)	−1.930 (Destabilizing)	−1.919 (Destabilizing)
4.	Sample 35	*MT-ATP8*	m.8420A > G	T19A	0.117 (Stabilizing)	−0.218 (Destabilizing)	−0.571 (Destabilizing)	1.980 (Stabilizing)	0.249 (Stabilizing)
5.	Sample 28	*MT-CO2*	m.7685A > G	I34V	−0.686 (Destabilizing)	−0.269 (Destabilizing)	−0.751 (Destabilizing)	−0.180 (Destabilizing)	−0.418 (Destabilizing)
6.	Sample 27,49	*MT-CO3*	m.9966G > A	V254I	0.408 (Stabilizing)	0.099 (Destabilizing)	−0.024 (Destabilizing)	0.270 (Stabilizing)	0.456 (Stabilizing)
7.	Sample 27,49	*MT-ND1*	m.4099C > T	L265F	−0.105 (Destabilizing)	0.005 (Stabilizing)	−0.67 (Destabilizing)	−0.57 (Destabilizing)	−0.711 (Destabilizing)
8.	Sample 50	MT-ND1	m.3392G > C	G29A	0.616 (Stabilizing)	0.084 (Stabilizing)	−0.887 (Destabilizing)	0.65 (Stabilizing)	−0.355 (Destabilizing)
9.	Samplse 50	*MT-ND1*	m.3460G > A	A52T	0.396 (Stabilizing)	0.136 (Destabilizing)	−0.989 (Destabilizing)	−1.330 (Destabilizing)	−0.870 (Destabilizing)
10.	Sample 3	*MT-ND2*	m.5279C > A	F270L	−1.182 (Destabilizing)	−0.362 (Destabilizing)	−1.748 (Destabilizing)	−1.53 (Destabilizing)	−1.871 (Destabilizing)
11.	Sample 40	*MT-ND2*	m.4638A > G	I57V	0.009 (Stabilizing)	−0.056 (Destabilizing)	−0.787 (Destabilizing)	−0.18 (Destabilizing)	−0.457 (Destabilizing)
12.	Sample 34	*MT-ND2*	m.5444C > A	F325L	−0.065 (Destabilizing)	0.027 (Stabilizing)	−0.396 (Destabilizing)	0.39 (Stabilizing)	−0.196 (Destabilizing)
13.	Sample 50	*MT-ND3*	m.10327C > T	S90L	0.375 (Stabilizing)	0.162 (Stabilizing)	−0.124 (Destabilizing)	1.310 (Stabilizing)	0.532 (Stabilizing)

### Interatomic interaction analysis

In the interatomic interaction analysis (Supplementary Figures 1–5), except for the m.8594 T > C variant, the two other *MT-ATP6* variants, m.9059C > T and m.9106A > G, exhibited stabilization through additional non-bonded interactions. No significant interatomic variations or alterations were detected for *MT-ATP8*, *MT-CO2* and *MT-CO3* variants.

### Haplogroup distribution

The phylogenetic analysis of mtDNA haplogroups in the LHON patient cohort revealed five distinct haplogroups (M, R, U, D, and W). The M haplogroup was the most prevalent (26/41, 63.4%), followed by R (9/41, 22.0%) and U (6/41, 14.6%). Haplogroups W and D each contributed one case.

## Discussion

In the current study, we analyzed the whole mitochondrial genome of 41 individuals with a clinical diagnosis of LHON who were negative for the three common mtDNA mutations based on conventional Sanger sequencing. Of these cases, NGS identified the m.11778G > A and m.3460G > A variant in one patient each, present in the heteroplasmic state (90.0 and 63.6%, respectively). A possible explanation for these mtDNA variants being missed could be the lower sensitivity of Sanger sequencing, which analyses a single DNA fragment, limiting its ability to detect heteroplasmic variants.

In our previous study (27), 82 out of 278 suspected LHON cases (29.4%) were found to be positive for one of the three primary mutations. This diagnostic rate is significantly lower than the figure of ~ 90% reported in other studies (6, 43), but it is comparable to findings from other studies of Indian populations (24, 26, 27). In this study, we explored the possible pathogenicity of other mtDNA variants in those patients who had tested negative for m.3460G > A, m.11778G > A and m.14484 T > C. We identified five previously reported LHON variants: three located in *MT-ND1* (m.3335 T > C, m.3394 T > C, m.3395A > G), one in *MT-ND4L* (m.10680G > A), and one in *MT-ND6* (m.14502 T > C).

### *MT-ND1* variants

The *MT-ND1* variants m.3335 T > C and m.3394 T > C were reported in a Chinese study involving 1,281 subjects. The m.3335 T > C variant was identified in four individuals with mild visual impairment. In contrast, the m.3394 T > C variant was found in 38 probands with varying degrees of visual impairment, accounting for an incidence rate of 2.97% (44). Another variant, m.3395A > G, was reported in a single suspected LHON (Leber’s Hereditary Optic Neuropathy) case from Argentina and has recently been reclassified from Likely Benign to Likely Pathogenic based on ACMG/AMP guidelines for mitochondrial DNA variant interpretation (45). The m.3394 T > C variant has also been reported in the Tibetan population, where it is associated with reduced complex I activity, influenced by mitochondrial haplogroup background and environment (46). It is notably enriched in the M9 haplogroup in Tibet. However, in our study, this variant was identified in the W3 haplogroup, which is relatively rare. The identification of this variant within W3, a lineage not typically associated with LHON, suggests that its pathogenic potential may vary by haplogroup. This highlights the influence of genetic background on disease expression. Importantly, no other rare variants were observed in the family, supporting a potential pathogenic role of this variant in the given haplogroup context. The *MT-ND1* polypeptide includes transmembrane, outer membrane, and intermembrane domains; notably, all three variants identified in this study are located within the transmembrane domain, which plays a critical role in mitochondrial complex I function (44).

### *MT-ND4L* and *MT-ND6* variants

The *MT-ND4L* variant m.10680G > A occurs at a highly conserved position, resulting in the amino acid change from alanine to threonine (A71T), with conservation rates of 86% in eukaryotes and 97% in mammals, and within an invariant stretch of 16 amino acids in primates (47). Studies of cybrid cell lines harboring this mtDNA variant demonstrated a respiration defect comparable to that observed in LHON cells (47). The variant has been identified in 14 haplogroups and has been previously reported as the sole pathogenic change in three LHON families, occurring as independent mutational events in haplogroups B4a1e, M13a1b, and D6a1 (48, 49). Additionally, this mutation has been found in association with the m.14484 T > C/*MT-ND6* mutation in another LHON family with a B4d1 haplogroup background (50). A separate family-based study also revealed the co-occurrence of three missense variants: m.10680G > A/*MT-ND4L*, m.12033A > G/*MT-ND4*, and m.14258G > A/*MT-ND6*, present in the proband, mother, and sister (47). The *MT-ND6* variant m.14502 T > C has been reported in three unrelated subjects from a Chinese LHON cohort, leading to the substitution of isoleucine for valine (I58V), a conserved amino acid across multiple species (51). Additionally, Zhang et al. (52) described four Chinese families in which individuals co-harboring both m.14502 T > C and m.11778G > A exhibited increased penetrance of visual loss, suggesting a potential synergistic effect. However, m.14502 T > C has also been identified at a relatively high frequency in population databases, including in healthy individuals, which challenges its role as a primary pathogenic LHON mutation. Given this, it is more plausible that m.14502 T > C serves as a secondary modifier or risk factor that may influence disease expression in the presence of other LHON-associated variants. Further functional and population-based studies are needed to clarify its exact role.

### *MT-CO3* variant

The missense variant m.9966G > A in *MT-CO3* is considered a helper variant, as it has been found to co-occur with LHON mtDNA variants (47) However, one Indian study identified this variant solely in two LHON families without any secondary variations (53). In the current study, we detected this variant in two families, Sample 27 and Sample 49, both belonging to the same haplogroup M38a. Interestingly, both families also carried the *MT-ND1* variant m.4099C > T, which was called as being deleterious, albeit only by CADD scoring. Additionally, Sample 49 carried a Variant of Unknown Significance (VUS) located in *MT-ATP6*, m.9106A > G, with very low frequencies in Mitomap (0.005%) and gnomAD (0.009%). Further evidence is needed to clarify the pathogenic role of the m.9966G > A variant in individuals diagnosed with LHON.

### Variants with conflicting interpretations

We identified two variants in *MT-ND1*: m.3316G > A in one family and m.4216 T > C in three different families. The pathogenicity of these variants remains unclear, with the reported incidences of these variants in a Chinese cohort being 4.0 and 1.09%, respectively (12). The m.3316G > A variant has been noted to co-occur with m.11778G > A mutations, and it has been suggested to increase the penetrance of LHON (12). We did not find any primary LHON mutations in the family associated with this variant in our cohort. The m.4216 T > C variant has been observed in both patient and control populations, and its pathogenic significance remains uncertain (12).

### Intermolecular interaction analysis

**Figure 5:**
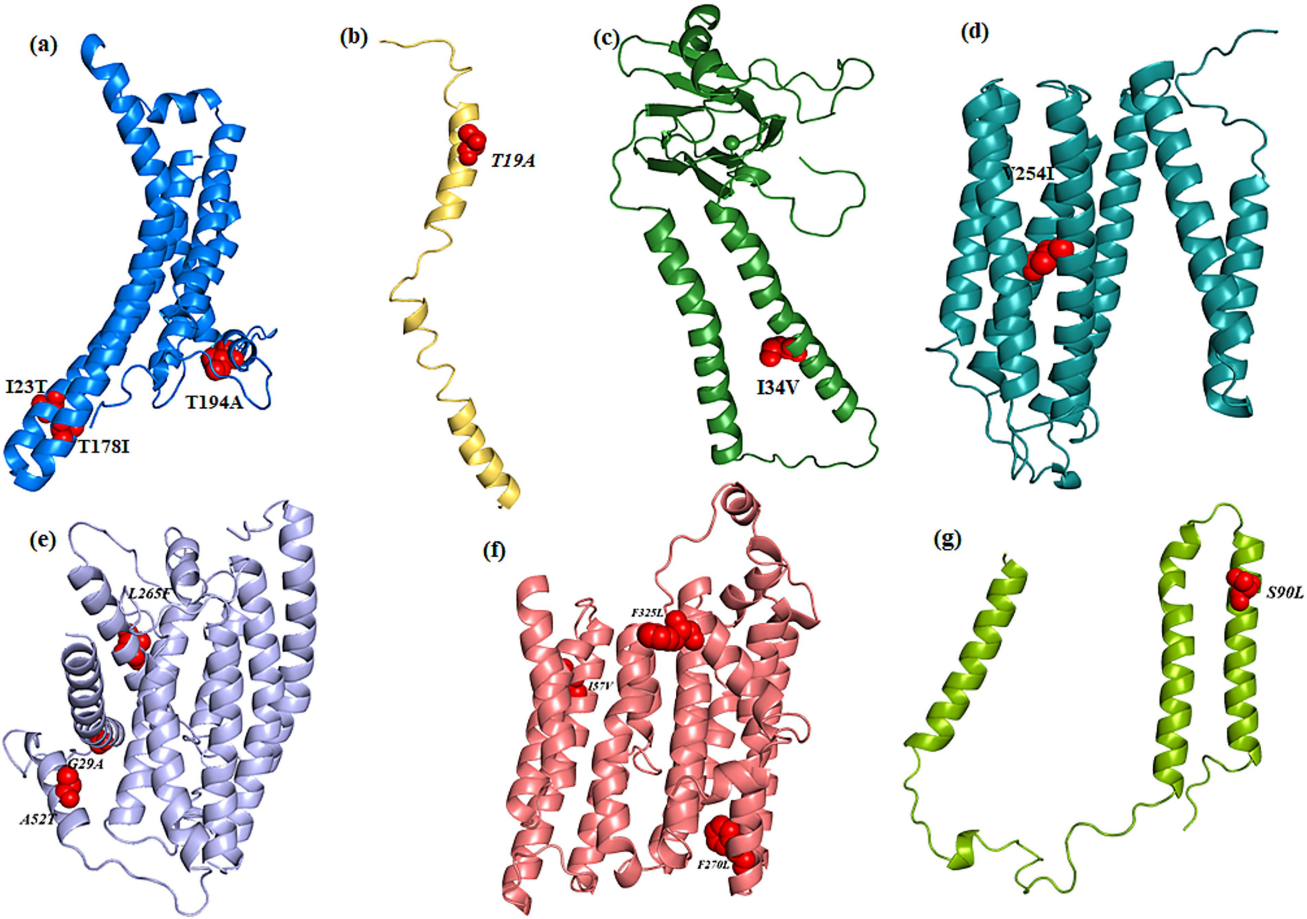
Cartoon representation of the modeled and refined structure of **(a)** MT*-ATP6*, **(b)** *MT-ATP8*, **(c)** *MT-COX2*, **(d)** *MT-COX3*, **(e)** *MT-ND1*, **(f)** *MT-ND2*, **(g)** *MT-ND3* wherein the variants are showed as spheres (red color).

The sequence-based pathogenicity analysis (Table 5) revealed that confirmed pathogenic LHON variants were found to be deleterious with high accuracy, primarily through MAPP prediction. All predictors identified the m.3460G > A variant in *MT-ND1* and the m.8594 T > C variant in *MT-ATP6* as highly deleterious. The presence of multiple variants, such as m.3460G > A and m.3392G > C could increase the pathogenicity score. Structural stability analysis of refined structures (Figure 5) showed that only the variants in *MT-ATP6*, *MT-ND2*, *MT-CO3*, and *MT-ND1* were associated with structural destabilization (Table 5). Specifically, the m.8594 T > C variant in *MT-ATP6*, m.5279C > A in *MT-ND2*, and m.7685A > G in *MT-CO2* negatively impacted their respective protein structures.

All the *MT-ATP6* variants maintained their interactions with the wild-type variant, except for m.8594 T > C (p. I23T), where T23 has lost its hydrophobic interaction with P12. The m.9059C > T (p. T178I) variant, I178, gained additional hydrophobic interactions with I193 and T189. Despite losing an H-bond with I191, the m.9106A > G (p. T194A) variant, which encodes A194, stabilizes *MT-ATP6* through strong van der Waals interactions with I192. In *MT-ATP8*, although the m.8420A > G (p. T19A) variant, A19, resulted in the loss of H-bonds with I23 and M16, all other interactions were preserved compared to the wild-type variant. For *MT-CO2*, the m.7685A > G (p. I34V) variant, V34 maintained H-bond interactions with I30 and V38 and gained an additional van der Waals interaction with I31. In *MT-CO3*, the m.9966G > A (p. V254I) variant, I254, did not induce interatomic changes, maintaining H-bond interactions with L250 and T259, and van der Waals interactions with L250, similar to the wild-type variant.

All the m.3392G > C, m.3460G > A, and m.4099C > T in *MT-ND1* maintained the interatomic interactions found with the wild-type variant. In *MT-ND2*, m.4638A > G (p. I57V), V57 formed van der Waals interactions with E54, differing from the wild type. Notably, variant 5279C > A (p. F270L), L270 lost hydrophobic interactions with Y196 and ionic interactions with M282.

For *MT-ND3*, the H-bond interactions were similar for the wild-type and the 10327C > T (p. S90L) variants. Functional impact analysis of all variants (Table 6) indicated that only the *MT-ND1* variant m.3392G > C (p. G29A) had a significant impact, such as alterations in disordered interfaces, ordered interfaces (Pr = 0.26 | *p* = 0.01), DNA binding, and transmembrane protein interactions (Table 7).

**Table 7:** Functional impact analysis of the identified mitochondrial DNA variants.

S. no	Sample ID	Gene	Variant	Amino acid	Functional/structural loss
1.	Sample 12	*MT-ATP6*	m.9059C > T	T178I	–
2.	Sample 49	*MT-ATP6*	m.9106A > G	T194A	–
3.	Sample 18, 44, 47	*MT-ATP6*	m.8594 T > C	I23T	–
4.	Sample 27, 49	*MT-ND1*	m.4099C > T	L265F	–
5.	**Sample 50**	***MT-ND1***	**m.3392G > C**	**G29A**	Altered Disordered interface (Pr = 0.28 | *p* = 0.04); Altered Ordered interface (Pr = 0.26 | *P* = 0.01); Altered DNA binding (Pr = 0.24 | *P* = 0.01); Altered Transmembrane protein (Pr = 0.20 | *p* = 5.2e-03)
6.	Sample 50	*MT-ND1*	m.3460G > A	A52T	–
7.	Sample 3	*MT-ND2*	m.5279C > A	F270L	–
8.	Sample 40	*MT-ND2*	m.4638A > G	I57V	–
9.	Sample 34	*MT-ND2*	m.5444C > A	F325L	–
10.	Sample 50	*MT-ND3*	m.10327C > T	S90L	–
11.	Sample 35	*MT-ATP8*	m.8420A > G	T19A	–
12.	Sample 28	*MT-CO2*	m.7685A > G	I34V	–
13.	Sample 27, 49	*MT-CO3*	m.9966G > A	V254I	–

The bold highlights the significant functional impact attained by the variant.

The analysis of missense mtDNA variants revealed a high proportion of variants that could be deleterious by disrupting protein structure and stability. Functional impact and conservation analyses further support the pathogenicity of several variants, particularly those in *MT-ATP6*, *MT-ND1*, and *MT-ND2*. Additionally, the co-occurrence of variants, such as those in *MT-ND1*, *MT-ATP6*, and *MT-CO3*, suggests potential synergistic effects on disease expression and severity, further complicating the interpretation of mitochondrial genetics in LHON. It has previously been reported that combinations of individually non-pathogenic missense mtDNA variants could cause low penetrance LHON (47).

Our findings underscore the complexity of LHON genetics, revealing not only known pathogenic mtDNA variants, but previously unreported variants that could play a role in disease pathogenesis. Future work is needed to determine whether a proportion of our undiagnosed LHON cohort carries nuclear genetic defects that are increasingly being recognized as an important cause of recessive LHON (2, 54).

### Overlapping clinical features suggest broader genetic etiology in LHON

All patients who presented with clinical features suggestive of Leber Hereditary Optic Neuropathy (LHON) but remained genetically unsolved (i.e., tested negative for known mitochondrial mutations) demonstrated a clinical profile similar to that of genetically confirmed (solved) LHON cases. The majority of patients in the unsolved group were male, with only one female patient identified. Most exhibited moderate vision loss without progression to severe visual impairment. Universally, patients in both cohorts showed temporal optic disk pallor at presentation, along with cecocentral visual field defects. No systemic manifestations were observed in either group.

MRI brain imaging revealed optic nerve signal changes in two patients from the solved group and one patient from the unsolved group. However, these findings were non-specific and not characteristic of LHON. Importantly, no distinct clinical or radiological differences were identified between the solved and unsolved cases. This clinical overlap underscores the need for further investigation, particularly nuclear gene analysis, with the help of Whole Exome Sequencing (WES) or Whole Genome Sequencing (WGS) to uncover the underlying genetic etiology in the unsolved cohort.

## Conclusion

A comprehensive analysis of the whole mitochondrial genome, preferably with NGS, is recommended in individuals with a clinical diagnosis of LHON in whom conventional Sanger sequencing has failed to uncover one of the three common mtDNA mutations. Additional research involving larger cohorts of Indian patients and complementary functional studies will be essential to validate our results and further clarify the full range of genetic contributors to LHON in this particular population.

## Data Availability

The datasets presented in this study can be found in online repositories. The names of the repository/repositories and accession number(s) can be found in the article/Supplementary material.

## Footnotes

^1^ http://www.mitomap.org/MITOMAP/MutationsLHON

^2^ http://doi.org/10.1093/nar/gkaa1032
