# Supplementary figures and images for: Whole mitochondrial genome sequencing in individuals with Leber hereditary optic neuropathy negative for the common pathogenic mitochondrial DNA variants
Source: Front Neurol. 2025 Sep 1;16:1584748. doi: 10.3389/fneur.2025.1584748 (PMC12442324; doi:10.3389/fneur.2025.1584748)

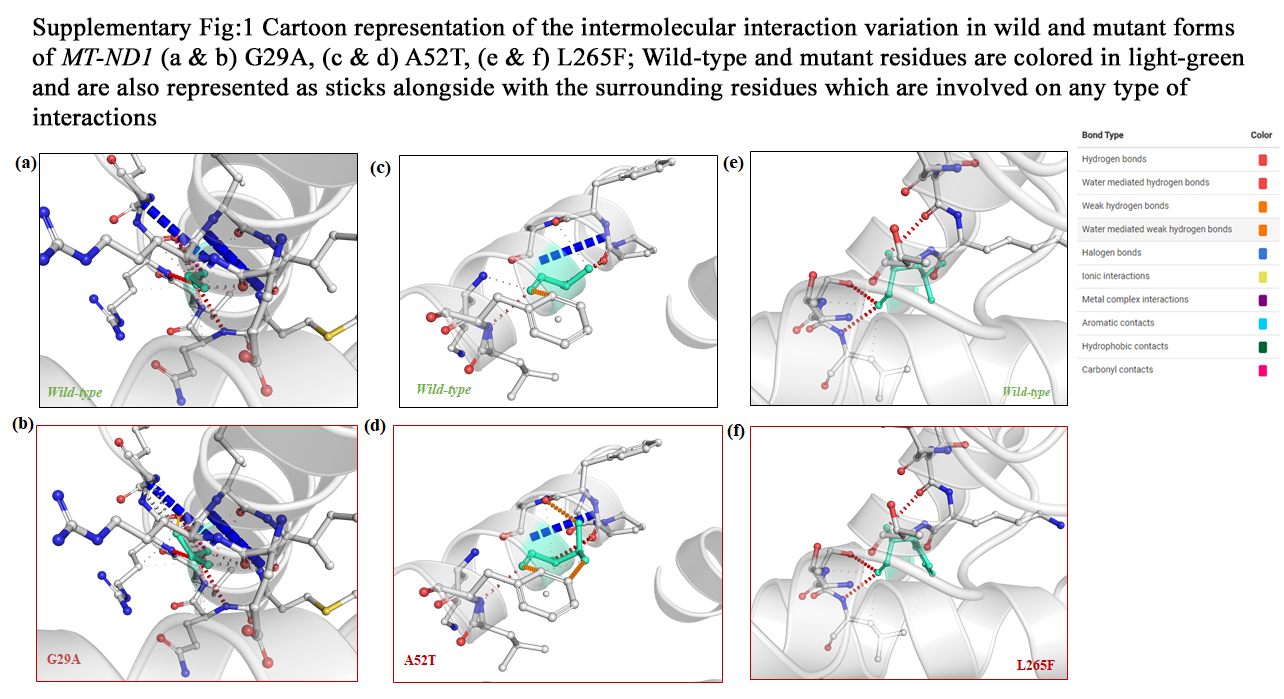

Supplement: Supplementary file 1 [file Image_1.TIF]

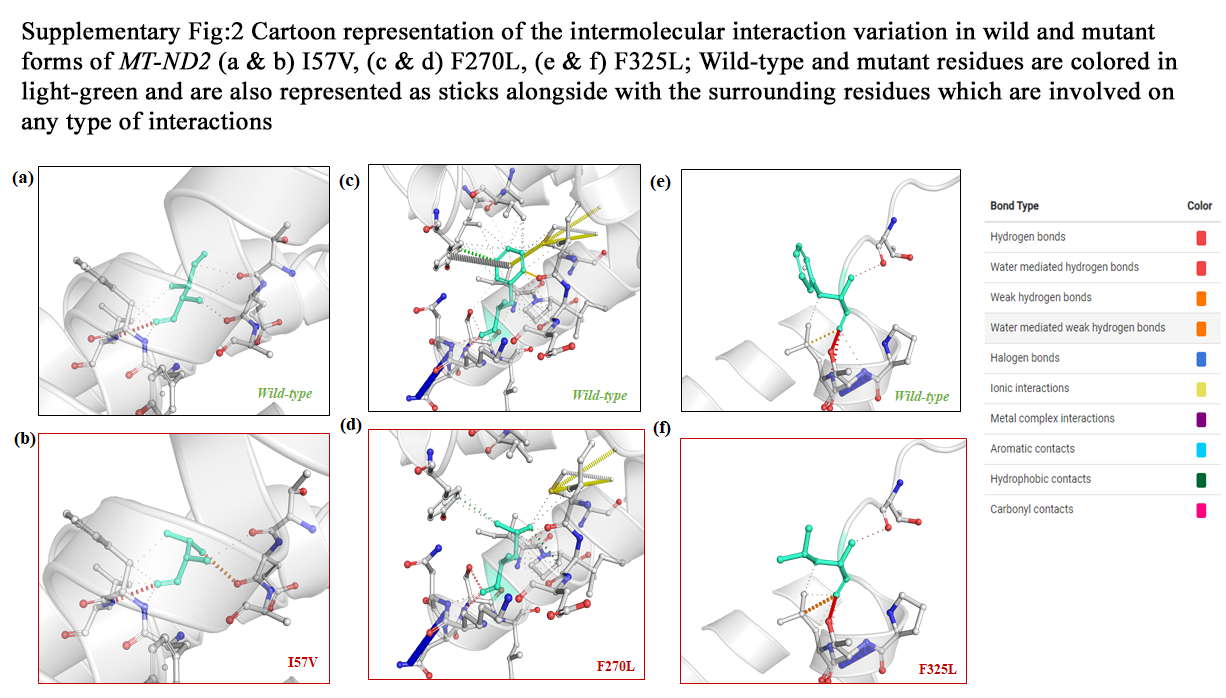

Supplement: Supplementary file 2 [file Image_2.TIF]

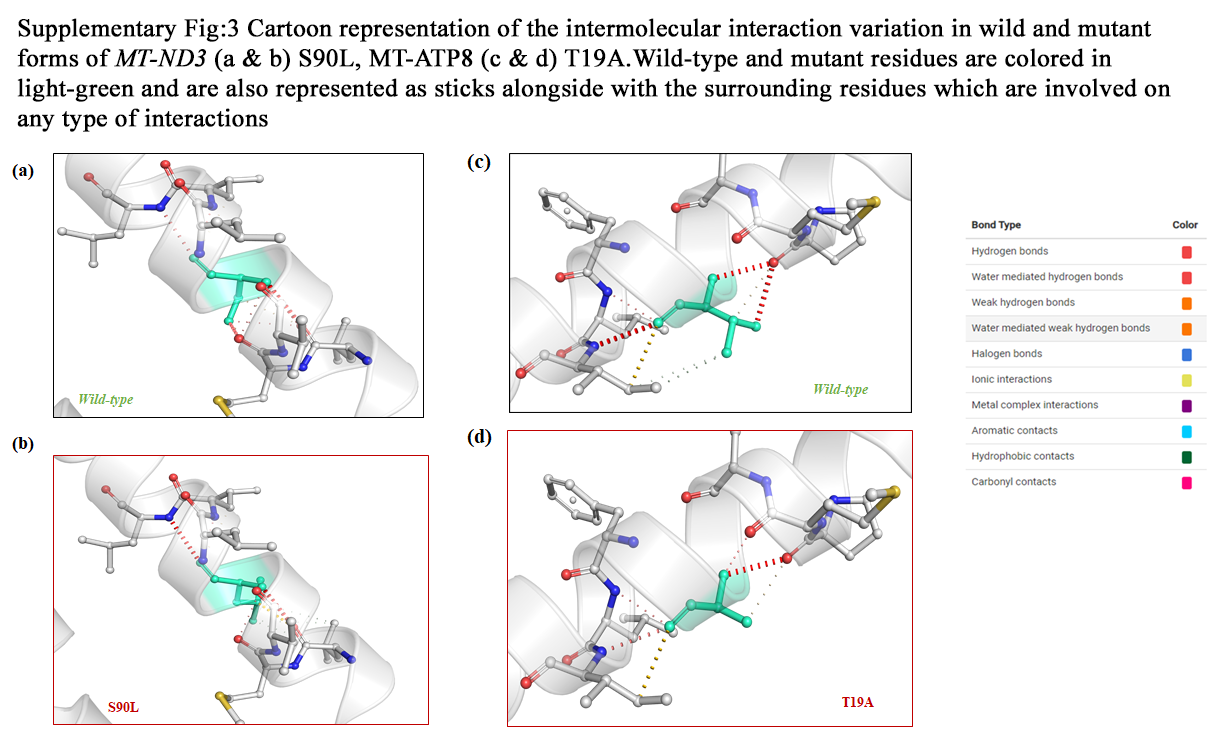

Supplement: Supplementary file 3 [file Image_3.TIF]

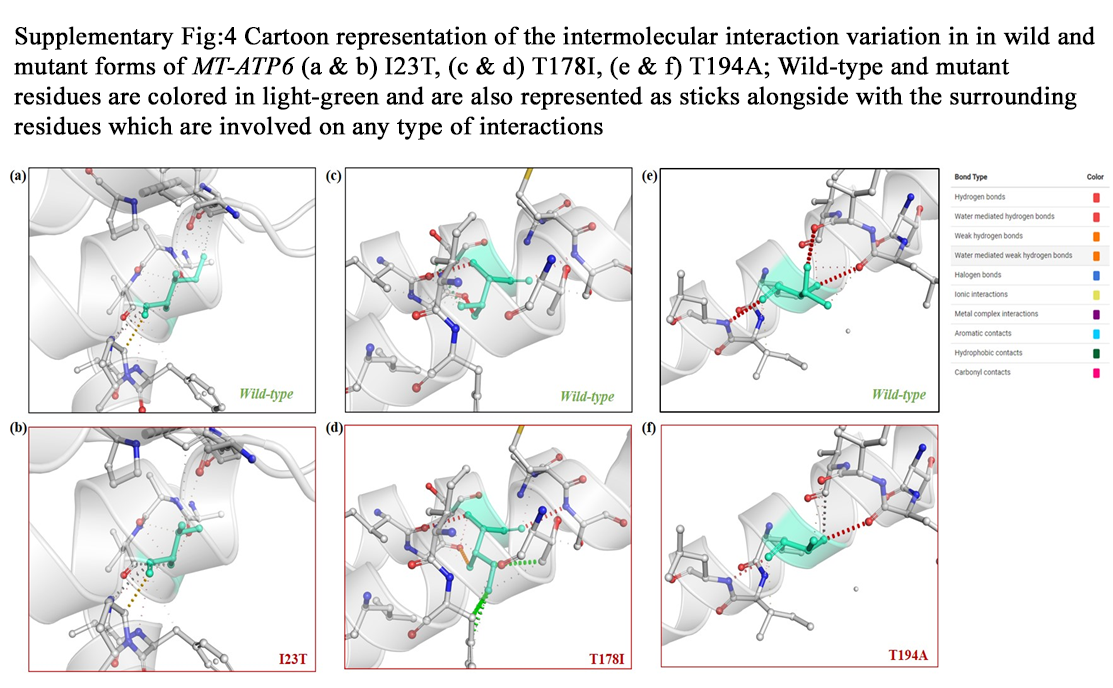

Supplement: Supplementary file 4 [file Image_4.TIF]

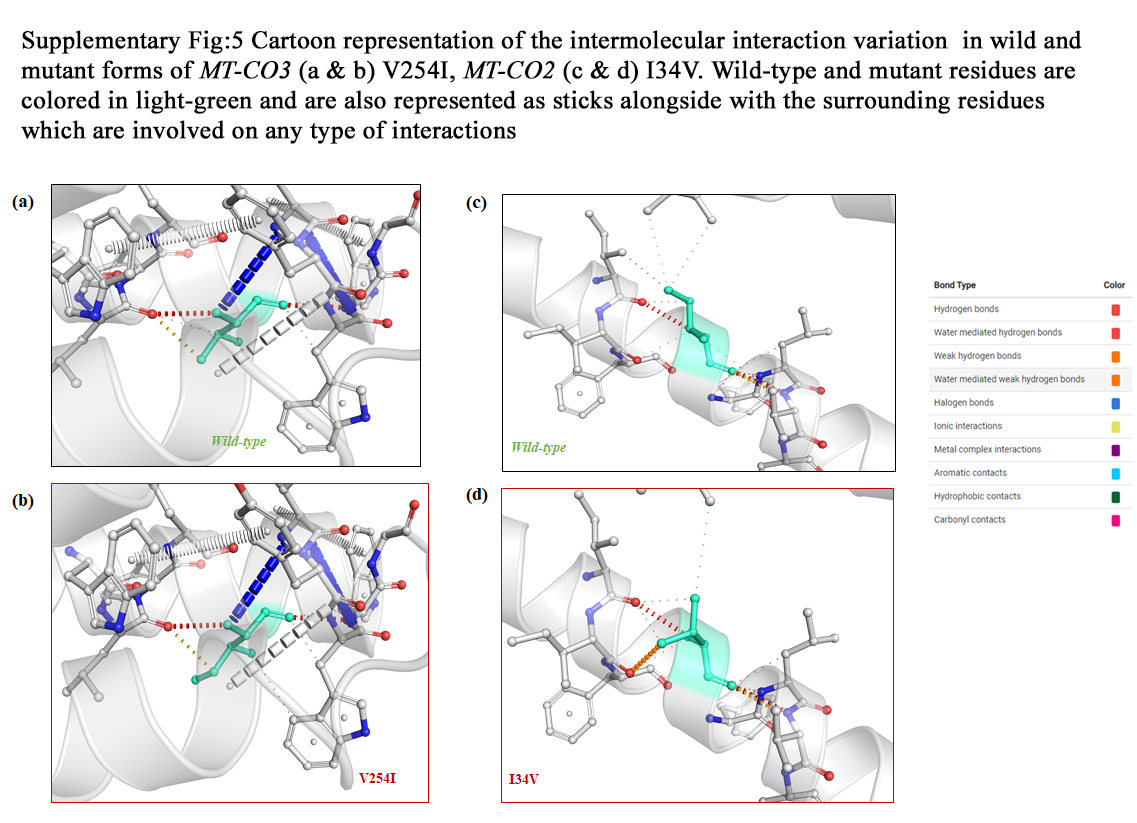

Supplement: Supplementary file 5 [file Image_5.TIF]
