# Supplementary material for: Whole mitochondrial genome sequencing in individuals with Leber hereditary optic neuropathy negative for the common pathogenic mitochondrial DNA variants
Source: Front Neurol. 2025 Sep 1;16:1584748. doi: 10.3389/fneur.2025.1584748 (PMC12442324; doi:10.3389/fneur.2025.1584748)
Supplement: Supplementary file 6 [file Table_1.docx]

| **S.No** | **Sample ID** | **Gene** | **Variant** | **Amino acid** | **PhyloP 100v Score** | **PhyloP 470way Score** | **PhastCons 100v Score** | **PhastCons 470way Score** |
| --- | --- | --- | --- | --- | --- | --- | --- | --- |
| 1. | Sample 50 | *MT-ND1* | m.3392G>C | G29A | 6.254 | 0.602 | 1 | 0.047 |
| 2. | Sample 50 | *MT-ND1* | m.3460G>A | A52T | 6.38 | 0.602 | 1 | 0.016 |
| 3. | Sample 27,49 | *MT-ND1* | m.4099C>T | L265F | -4.319 | 0.353 | 0 | 0.011 |
| 4. | Sample 40 | *MT-ND2* | m.4638A>G | I57V | 3.501 | 0.58 | 0.098 | 0.003 |
| 5. | Sample 34 | *MT-ND2* | m.5444C>A | F325L | -9.083 | -0.552 | 0 | 0 |
| 6. | Sample 3 | *MT-ND2* | m.5279C>A | F270L | -4.278 | -0.812 | 0 | 0.006 |
| 7. | Sample 50 | *MT-ND3* | m.10327C>T | S90L | 0.675 | -0.441 | 0 | 0 |
| 8. | Sample 35 | *MT-ATP8* | m.8420A>G | T19A | -0.727 | -0.816 | 0 | 0 |
| 9. | Sample18, 44, 47 | *MT-ATP6* | m.8594T>C | I23T | 4.715 | 0.361 | 0.988 | 0.074 |
| 10. | Sample 12 | *MT-ATP6* | m.9059C>T | T178I | 0.839 | -0.162 | 0 | 0.003 |
| 11. | Sample 49 | *MT-ATP6* | m.9106A>G | T194A | -0.633 | -0.176 | 0 | 0.002 |
| 12. | Sample 27,49 | *MT-CO3* | m.9966G>A | V254I | 0.072 | 0.848 | 0.006 | 0.964 |
| 13. | Sample 28 | *MT-CO2* | m.7685A>G | I34V | 7.067 | 0.819 | 1 | 0.12 |

**Supplementary Table 1. Conservation analysis of all the variants using MitImpact 3D**
