# Supplementary material for: Whole mitochondrial genome sequencing in individuals with Leber hereditary optic neuropathy negative for the common pathogenic mitochondrial DNA variants
Source: Front Neurol. 2025 Sep 1;16:1584748. doi: 10.3389/fneur.2025.1584748 (PMC12442324; doi:10.3389/fneur.2025.1584748)
Supplement: Supplementary file 7 [file Table_2.docx]

**Supplementary Table 2 . Pathogenicity prediction by Meta-predictors**

| **Sample ID** | **Gene** | **Variant** | **APOGEE1** | **APOGEE2** | **CAROL** | **Condel** | **COVEC WMV** | **MtoolBox** | **DEOGEN2** | **Meta SNP** |
| --- | --- | --- | --- | --- | --- | --- | --- | --- | --- | --- |
| *MT-ND1* | m.3392G>C | G29A | Pathogenic | Likely-pathogenic | Deleterious | Neutral | Deleterious | Deleterious | Tolerated | . |
| *MT-ND1* | m.3460G>A | A52T | Pathogenic | Likely-pathogenic | Deleterious | Neutral | Deleterious | Deleterious | Tolerated | Disease |
| *MT-ND1* | m.4099C>T | L265F | Neutral | Likely-benign | Neutral | Deleterious | Neutral | Neutral | Tolerated | . |
| *MT-ND2* | m.4638A>G | I57V | Neutral | Benign | Neutral | Deleterious | Neutral | Neutral | Tolerated | . |
| *MT-ND2* | m.5444C>A | F325L | Neutral | Benign | Neutral | Deleterious | Neutral | Neutral | Tolerated | . |
| *MT-ND2* | m.5279C>A | F270L | Neutral | Benign | Neutral | Deleterious | Neutral | Neutral | Tolerated | . |
| *MT-ND3* | m.10327C>T | S90L | Neutral | Benign | Neutral | Deleterious | Neutral | Neutral | Tolerated | . |
| *MT-ATP8* | m.8420A>G | T19A | Neutral | Benign | Neutral | Neutral | Neutral | Deleterious | Tolerated | . |
| *MT-ATP6* | m.8594T>C | I23T | Neutral | Likely-benign | Neutral | Deleterious | Neutral | Deleterious | Tolerated | . |
| *MT-ATP6* | m.9059C>T | T178I | Neutral | Likely-benign | Neutral | Deleterious | Neutral | Deleterious | Tolerated | . |
| *MT-ATP6* | m.9106A>G | T194A | Neutral | Neutral | Deleterious | Neutral | Neutral | Deleterious | Tolerated | . |
| *MT-CO3* | m.9966G>A | V254I | Neutral | Benign | Neutral | Deleterious | Neutral | Neutral | Tolerated | . |
| *MT-CO2* | m.7685A>G | I34V | Pathogenic | Likely-benign | Neutral | Deleterious | Neutral | Neutral | Tolerated | . |
